# Hyperthyroidism Due to Functioning Metastatic Bone Lesions of Follicular Thyroid Carcinoma Treated With Lenvatinib

**DOI:** 10.1210/jcemcr/luae139

**Published:** 2024-07-24

**Authors:** Tomoko Kobayashi, Shintaro Iwama, Koji Suzuki, Hiroshi Arima

**Affiliations:** Department of Endocrinology and Diabetes, Nagoya University Graduate School of Medicine, Nagoya 466-8550, Japan; Department of Endocrinology and Diabetes, Nagoya University Graduate School of Medicine, Nagoya 466-8550, Japan; Department of Endocrinology and Diabetes, Nagoya University Graduate School of Medicine, Nagoya 466-8550, Japan; Department of Endocrinology and Diabetes, Nagoya University Graduate School of Medicine, Nagoya 466-8550, Japan

**Keywords:** thyrotoxicosis, functioning metastases, follicular thyroid carcinoma, lenvatinib

## Abstract

A 71-year-old woman was diagnosed with unresectable metastatic follicular thyroid carcinoma (FTC) and thyrotoxicosis. She was negative for the presence of thyroxine receptor antibody and thyroid-stimulating antibody. Whole-body scintigraphy revealed increased ^99m^Tc-pertechnetate uptake in metastatic bone lesions but not in the thyroid nodule. Since radioactive iodine therapy was not applicable because the canalis vertebralis had been invaded, treatment with lenvatinib was initiated, along with methimazole and potassium iodide. The serum level of thyroid hormone decreased. The patient developed hypothyroidism, which continued after the methimazole was stopped, suggesting that lenvatinib suppressed the hyperthyroidism. To our best knowledge, this is the first report of a patient with functioning bone lesions of metastatic FTC in whom hyperthyroidism was controlled by lenvatinib without radioactive iodine therapy.

## Introduction

The differential diagnosis of hyperthyroidism includes thyroiditis, Graves disease, toxic multinodular goiter, and struma ovarii [1]. Hyperthyroidism due to thyroid carcinoma is a rare entity that also presents a therapeutic challenge, since it is not easy to control with antithyroid agents. In this situation, radioiodine therapy usually provides prompt improvement in thyroid hyperfunctioning [2]. Herein, we present an unusual case of hyperthyroidism due to functioning metastatic bone lesions of follicular thyroid carcinoma (FTC), which were identified by their increased uptake of ^99m^Tc-pertechnetate on whole-body scintigraphy. The hyperthyroidism was effectively controlled by lenvatinib together with antithyroid agents.

## Case Presentation

**Figure 1.**
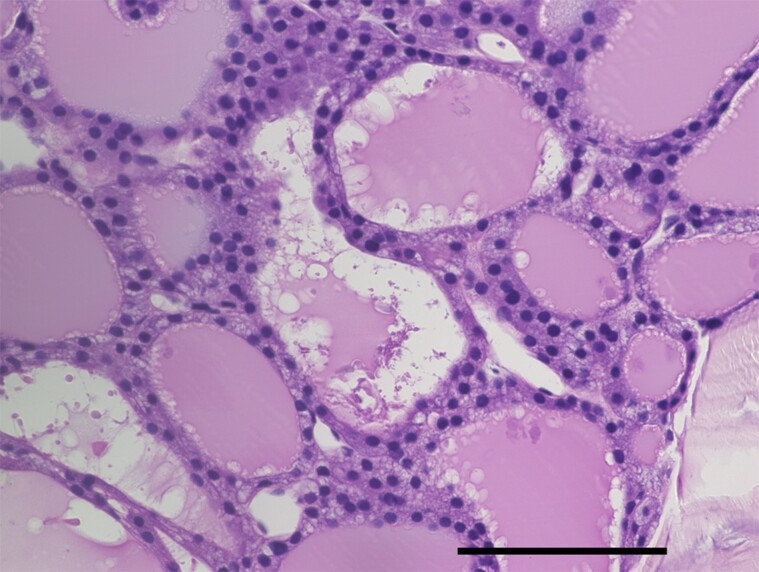
Histopathological examination of a section of a biopsy specimen from the lesion in the right iliac bone revealed atypical follicular epithelial cells with mildly enlarged nuclei forming follicular structures. Numerous vacuoles are present just inside the follicular epithelial cells of the thyroid. Scale bar indicates 100 µm.

The patient was a 71-year-old woman, who did not have a significant medical or family history and had had back pain for a year. She visited a local clinic because her back pain had worsened. She was diagnosed with compression fractures of her lumbar vertebrae (L1-L3) and was referred to an orthopedic specialist. Computed tomography (CT) revealed lesions in her sternum (largest diameter, 46 mm), L2 vertebral arch (67 mm), sacrum (39 mm), and ilium (64 mm), indicating widespread bone metastases. Histopathology of the right ilium biopsy demonstrated atypical follicular epithelial cells with mildly enlarged nuclei that had infiltrated and proliferated within the trabecular spaces and had formed follicular structures (Fig. 1). Immunohistochemical testing revealed positive staining for thyroid transcription factor-1, cytokeratin 7, and paired box protein PAX-8, which are known to be expressed in thyroid follicular carcinoma [3]. These findings, along with the morphological identification of thyroid follicular structures, were diagnosed as FTC metastatic to the bones. Ultrasonography revealed a calcified 14-mm nodule in the right lobe of the thyroid gland without any local invasion. A fine-needle aspirate of the nodule was diagnosed as a thyroid follicular neoplasm. The patient was hospitalized to receive treatment for metastatic FTC.

## Diagnostic Assessment

Physical examination on hospital admission revealed a body temperature of 37.3 °C, pulse of 81 beats/min, and blood pressure of 155/74 mm Hg. The patient had noticed elevated blood pressure over the past month, but no weight loss, palpitations, or tremor. Examination of the neck was negative for goiter and tenderness. A hard, palpable 6-cm mass on her sternum was noted. Routine hematological testing was within normal limits. Thyroid function testing showed elevated levels of free tri-iodothyronine (FT3) and free thyroxine (FT4), and undetectable thyroxine (TSH) (Table 1). The patient's serum thyroglobulin (Tg) and antithyroglobulin antibody (TgAb) level were elevated (see Table 1). Tests for TSH receptor antibody (TRAb), thyroid-stimulating antibody (TSAb), and antithyroperoxidase antibody were negative.

**Table 1.** Thyroid function testing data of the patient on admission

	Data tested	Normal range
FT3	17.60 pg/mL (27.10 pmol/L)	1.68-3.67 pg/mL (2.59-5.65 pmol/L)
FT4	2.50 ng/dL (32.25 pmol/L)	0.70-1.48 ng/dL (9.03-19.09 pmol/L)
TSH	<0.009 µIU/mL (<0.009 mIU/L)	0.35-4.94 µIU/mL (0.35-4.94 mIU/L)
Tg	80 590 ng/mL (80 590 µg/L)	0-33.7 ng/mL (0-33.7 µg/L)
TgAb	629 IU/mL	<28 IU/mL

Values in parentheses are International System of Units (SI).

Abbreviations: FT3, free tri-iodothyronine; FT4, free thyroxine; Tg, thyroglobulin; TgAb, antithyroglobulin antibody; TSH, thyroxine.

**Figure 2.**
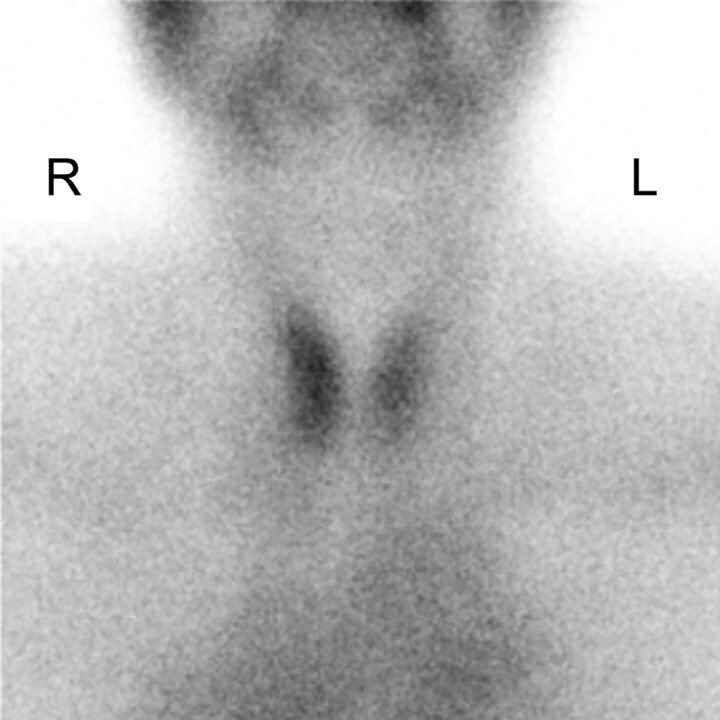
A ^99m^Tc-pertechnetate scintigram on admission shows normal uptake in the thyroid gland. L, left; R, right.

Tg, TgAb, TRAb, and antithyroperoxidase antibody levels were measured by electrochemiluminescent immunoassays (Elecsys Tg II kit, Elecsys Anti-Tg kit, Elecsys Anti-TSHR kit, and Elecsys Anti-TPO kit, respectively; Roche Diagnostics). Tg values can be affected by the presence of TgAb, which would lead to falsely low Tg values [4]. The TSAb level was measured by an enzyme immunoassay (TSAb kit, Yamasa Bioassay EIA; YAMASA Corp). A ^99m^Tc-pertechnetate scintigram, which can be readily performed without iodine restriction, showed low-normal uptake in the thyroid gland (0.6%, normal range, 0.4%-3.0%) (Fig. 2). At the time this test was performed, the patient's thyrotoxicosis was thought to be due to destructive thyroiditis.

## Treatment

The option of radioactive iodine therapy following thyroidectomy was ruled out for this patient with an invasive metastatic tumor of the canalis vertebralis, because of the potential risk of radiation-induced edema of the spinal cord [5, 6]. Instead, palliative external radiation therapy (30 Gy/10 fractions/10 days) was administered to the metastatic bone lesions at L1 to L3. For systemic therapy, lenvatinib (24 mg/day) was initiated on day 0, but it was discontinued on day 6 because the patient had developed fatigue, high blood pressure, loss of appetite, nausea, and mild diarrhea. While her pedal edema had persisted from the time of admission, no other common side effects of lenvatinib such as palmar-plantar erythrodysesthesia, skin rash, hyponatremia, vomiting, abdominal pain, or constipation were observed. β-Blockers were administered to treat the patient's thyrotoxicosis.

**Figure 3.**
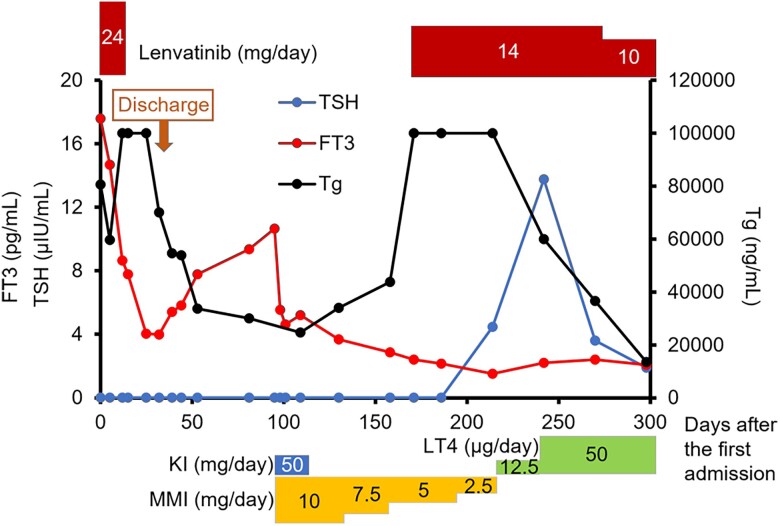
Graph showing the patient's clinical course and treatment. Changes in serum levels of FT3, TSH and Tg are depicted. The dosages of drugs administered to the patient are shown at the top and the bottom. Conversion factors for SI and conventional units are FT3: 1.0 pg/mL = 1.54 pmol/L, TSH: 1.0 µIU/mL = 1.0 mIU/L, and Tg: 1.0 µg/L = 1.0 ng/mL. FT3, free tri-iodothyronine; KI, potassium iodine; LT4, levothyroxine; MMI, methimazole; Tg, thyroglobulin; TSH, thyroxine.

**Figure 4.**
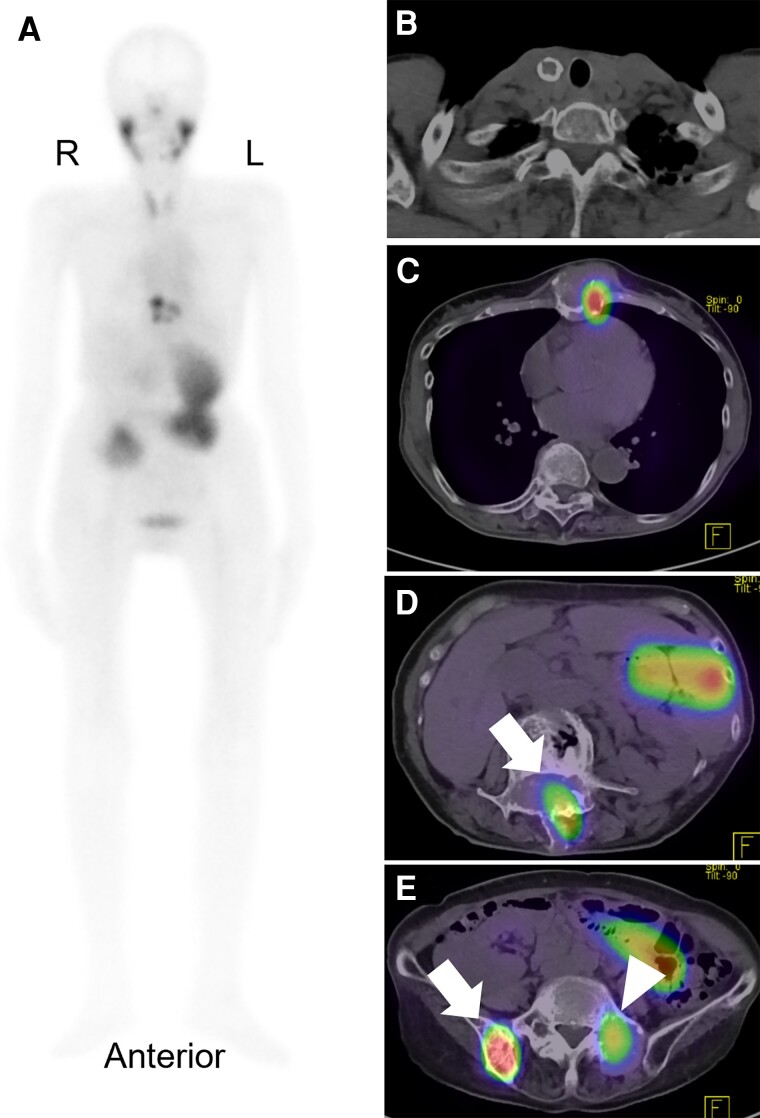
A, Whole-body ^99m^Tc-pertechnetate scintigram obtained at the recurrence of thyrotoxicosis. B, Although uptake at the thyroid bed was at the lower limit of normal, increased uptake is shown for the C, sternum; D, lumbar spine (white arrow); E, sacrum (white arrowhead); and E, right ilium (white arrow), despite decreased thyrotropin levels. L, left; R, right.

Since her symptoms had improved, the patient was scheduled for discharge; however, it was postponed because of a trochanteric fracture of the femur when she fell on day 18. On day 32, her FT3 and FT4 serum levels decreased to 3.99 pg/mL (6.14 pmol/L) and 0.81 ng/dL (10.45 pmol/L), respectively (Fig. 3). The patient was discharged on day 46; however, thyrotoxicosis was again detected biochemically by thyroid function testing on day 53 (see Fig. 3). The patient was again negative for the presence of TRAb and TSAb. Whole-body ^99m^Tc-pertechnetate scintigraphy on day 91 revealed increased uptake in the sternum, lumbar spine, sacrum, and right ilium (total, 13.1%), while the uptake at the thyroid bed was 0.4% (Fig. 4A-4E). Based on these results, we concluded that the cause of thyrotoxicosis in this patient was due to functioning metastatic bone lesions of FTC.

## Outcome and Follow-up

**Figure 5.**
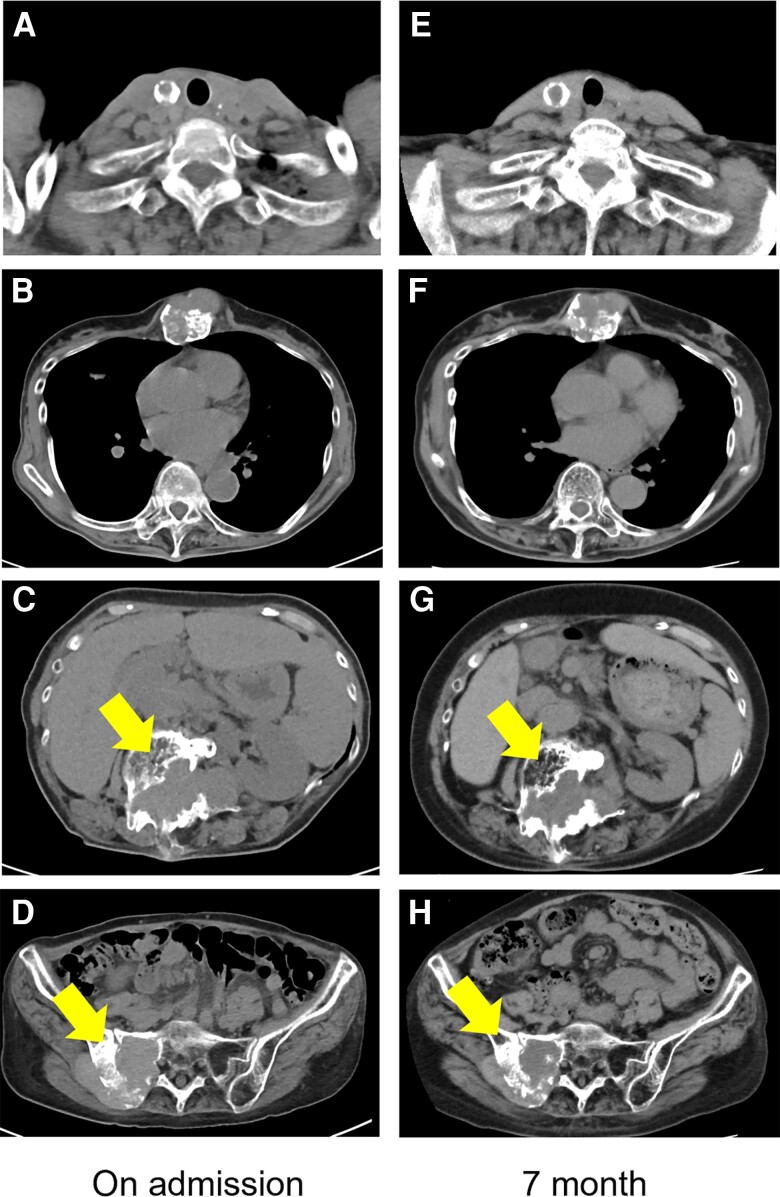
Comparison of computed tomography scans of the lesions in the A and E, thyroid; B and F, sternum; C and G, lumbar spine; and D and H, sacrum. There were no marked changes in the respective sizes of each lesion between A to D, admission and E to H, 7 months after admission.

Thiamazole (10 mg/day) and potassium iodide (50 mg/day) were initiated for the patient's hyperthyroidism on day 95. On day 102, after her FT3 and FT4 levels had dropped, potassium iodide was stopped followed by the tapering of thiamazole (see Fig. 3). With the cessation of lenvatinib treatment on day 6, the patient's serum Tg levels gradually decreased until day 109, and then started to increase thereafter. Lenvatinib was resumed at a reduced dosage of 14 mg/day on day 171, with a subsequent decrease in serum Tg levels. These findings indicated that antitumor effects were obtained, although there were no changes in the sizes of the metastatic lesions (Fig. 5). The nadir levels of Tg and TgAb were 13 136 ng/mL (13 136 µg/L) on day 300 and 61.6 IU/mL on day 298, respectively. Since the patient's serum TSH levels had increased (4.48 µIU/mL [4.48 mIU/L]), even after discontinuation of thiamazole on day 214 (43 days after the resumption of lenvatinib treatment), levothyroxine administration was initiated in increasing doses to a final dosage of 50 µg/day (see Fig. 3).

## Discussion

Hyperthyroidism due to FTC is rare. A previous study showed that 19 of 924 (2.1%) cases of differentiated thyroid carcinoma were identified as hyperfunctional, and 15 of those carcinomas were identified as FTCs [7]. Another study of iodine deficiency reported that in 422 patients with thyroid carcinoma, the incidence of hyperthyroidism other than Graves disease was 2.8% (n = 12) and the incidence of hot nodules on thyroid scintigraphy was 1.2% (n = 5) [8].

Possible mechanisms of hyperthyroidism in patients with thyroid carcinoma include somatic mutations in the gene for thyrotropin receptor (*TSHR*) [9] or mutations in the *PRKAR1A* gene [10]. Another mechanism could be stimulation of the TSH receptors by TSAb [11], although the patient was negative both for TRAb and TSAb.

Notably, our patient's metastatic bone lesions showed increased uptake of ^99m^Tc-pertechnetate on scintigraphy, whereas her primary thyroid lesion did not show increased uptake. A systematic review reported 6 patients only with thyroid tumors without increased uptake on scans of the thyroid, who had distant metastatic lesions showing high uptake on whole-body scans [12]. The reasons accounting for the absence of substantial uptake in the thyroid area but the presence of elevated uptake in distant metastatic lesions on whole-body scans remain unverified. However, the findings might be accounted for by the differences between tumor volumes and functional activities. The volumes of tumor in the metastatic bone lesions (range of largest diameters, 39-67 mm) were much larger than the volume of the primary lesion (largest diameter: 14 mm), which might have resulted in different degrees of active synthesis of thyroid hormone. The histopathological findings that there were numerous vacuoles in the follicles of our patient's metastatic lesion also indicated that our patient's metastatic lesions showed elevated degrees of the synthesis of thyroid hormones.

Another explanation for the differences between degrees of ^99m^Tc-pertechnetate uptake is that metastases in large bones may show phenotypic changes that would produce more thyroid hormones compared to the primary thyroid lesion. Sundaraiya et al [13] suggested that changes in the expression of sodium iodide symporter, an integral plasma membrane glycoprotein that mediates the thyroidal uptake of ^99m^Tc-pertechnetate, could explain the differences between the findings of ^99m^Tc-pertechnetate scintigraphy for the primary thyroid lesion and the metastatic lesions.

The treatment for most patients with differentiated thyroid carcinoma consists of thyroidectomy followed by thyroid hormone replacement and selective use of radioiodine therapy [14]. Some patients with differentiated metastatic thyroid carcinoma can be cured by radioiodine therapy, and treatments with thyroid hormone that suppress TSH levels can help slow disease progression [14]. Other treatment options include systemic chemotherapies that include multikinase inhibitors such as lenvatinib [15], and external radiotherapy for palliation of locally advanced, unresectable metastatic lesions [14]. Our patient was treated by lenvatinib and external radiotherapy because radioactive iodine therapy following total thyroidectomy was not applicable since the canalis vertebralis had been invaded. However, if lenvatinib treatment leads to marked shrinkage of the invasive tumor of the canalis vertebralis, radioactive iodine therapy following thyroidectomy may be possible.

Although a temporary decrease in the serum levels of FT3 and FT4 was observed after the first administration of lenvatinib, it was not possible to determine whether this was due to lenvatinib because it was administered for 6 days only. However, because hypothyroidism developed 43 days after the resumption of lenvatinib, it was thought that our patient's hypothyroidism was induced by lenvatinib. The effect of external radiotherapy on thyroid hormone control could be small if any, because the irradiated focus (L1-L3) was a small part of widespread metastatic lesions.

A phase 3 clinical trial of patients with unresectable hepatocellular carcinoma that compared the efficacy and safety of lenvatinib and sorafenib only without surgery or radiotherapy found a 16.4% incidence of hypothyroidism associated with lenvatinib [16]. A subanalysis of a Japanese population showed that the incidence of hypothyroidism induced by lenvatinib was 40.7% [17]. Another real-world study of patients with unresectable hepatocellular carcinoma who received lenvatinib alone without surgery or radiotherapy found an incidence of 52.0% of overt hypothyroidism associated with lenvatinib [18]. Plausible explanations for this result are that lenvatinib triggers autoimmunity [19, 20] or reduces the vascular density and fenestrations of the thyroid via inhibition of the activities of the vascular endothelial growth factor receptor. Therefore, the uptake of iodine and production of thyroid hormones by the gland is reduced [21].

Besic and Vidergar-Kralj [22] reported a patient with metastatic oncocytic carcinoma of the thyroid who developed hyperthyroidism after total thyroidectomy for Graves disease, in whom lenvatinib was effective for the treatment of hyperthyroidism. Their report suggests that lenvatinib might be an option for controlling hyperthyroidism due to functioning metastatic lesions. To our best knowledge, our patient with FTC is the first patient case of hyperthyroidism due to functioning metastatic bone lesions that was controlled by lenvatinib. The outcome suggests that early treatment by lenvatinib may be effective for controlling hyperthyroidism due to functioning FTC, as evidenced by the relatively prompt decreases in serum thyroid hormones associated with lenvatinib treatment.

In summary, we report an unusual case of a patient with hyperthyroidism due to functioning metastatic bone lesions of FTC, which was managed by lenvatinib and antithyroid drugs. This case also emphasizes the importance of this entity in the differential diagnosis of a patient with metastatic thyroid cancer who presents with thyrotoxicosis.

## Learning Points

- Clinicians should be aware that hyperthyroidism could occur because of functioning metastatic lesions of thyroid carcinoma.
- Consider performing whole-body thyroid scintigraphy for hyperthyroidism patients with metastatic lesions from thyroid carcinoma.
- Hyperthyroidism due to functioning thyroid carcinoma may be controlled by lenvatinib.

## Contributors

All authors made individual contributions to authorship. T.K., S.I., and H.A.: diagnosis and management of the patient. T.K., S.I., and K.S.: preparation of the histology image. T.K. and K.S.: responsible for editing of clinical images, investigation results, and drawing original diagrams. T.K., S.I., and H.A.: responsible for drafting of the text, and sourcing and critical revision for important intellectual content. All authors reviewed and approved the final draft.

## Data Availability

Some or all data sets generated during and/or analyzed during the current study are not publicly available but are available from the corresponding author on reasonable request.

## Abbreviations

FT3: free tri-iodothyronine
FT4: free thyroxine; FTC = follicular thyroid carcinoma
Tg: thyroglobulin
TgAb: antithyroglobulin antibody
TRAb: TSH receptor antibody
TSAb: thyroid-stimulating antibody
TSH: thyroxine
